# Modeling DNA Methylation Profiles through a Dynamic Equilibrium between Methylation and Demethylation

**DOI:** 10.3390/biom10091271

**Published:** 2020-09-03

**Authors:** Giulia De Riso, Damiano Francesco Giuseppe Fiorillo, Annalisa Fierro, Mariella Cuomo, Lorenzo Chiariotti, Gennaro Miele, Sergio Cocozza

**Affiliations:** 1 Dipartimento di Medicina Molecolare e Biotecnologie Mediche, Università degli Studi di Napoli “Federico II”, Via S. Pansini 5, 80131 Naples, Italy; mariella.cuomo@unina.it (M.C.); chiariot@unina.it (L.C.); sergio.cocozza@unina.it (S.C.); 2 Dipartimento di Fisica “E. Pancini”, Università degli Studi di Napoli “Federico II”, 80126, Naples, Italy; dfgfiorillo@na.infn.it (D.F.G.F.); miele@na.infn.it (G.M.); 3 Istituto Nazionale di Fisica Nucleare, Sezione di Napoli, 80126 Naples, Italy; 4 CNR-SPIN, c/o Complesso di Monte S. Angelo, via Cinthia, 80126 Naples, Italy; annalisa.fierro@spin.cnr.it; 5 CEINGE Biotecnologie Avanzate, via Gaetano Salvatore 482, 80145 Naples, Italy

**Keywords:** DNA methylation, DNA demethylation, mathematical modeling, statistical equilibrium, cell-to-cell heterogeneity, methylation profiles, epialleles

## Abstract

DNA methylation is a heritable epigenetic mark that plays a key role in regulating gene expression. Mathematical modeling has been extensively applied to unravel the regulatory mechanisms of this process. In this study, we aimed to investigate DNA methylation by performing a high-depth analysis of particular loci, and by subsequent modeling of the experimental results. In particular, we performed an in-deep DNA methylation profiling of two genomic loci surrounding the transcription start site of the D-Aspartate Oxidase and the D-Serine Oxidase genes in different samples (n = 51). We found evidence of cell-to-cell differences in DNA methylation status. However, these cell differences were maintained between different individuals, which indeed showed very similar DNA methylation profiles. Therefore, we hypothesized that the observed pattern of DNA methylation was the result of a dynamic balance between DNA methylation and demethylation, and that this balance was identical between individuals. We hence developed a simple mathematical model to test this hypothesis. Our model reliably captured the characteristics of the experimental data, suggesting that DNA methylation and demethylation work together in determining the methylation state of a locus. Furthermore, our model suggested that the methylation status of neighboring cytosines plays an important role in this balance.

## 1. Introduction

DNA methylation is a heritable epigenetic mark, consisting of the covalent binding of a methyl group to the 5th carbon of cytosines. In mammals, this modification mainly occurs at CpG dinucleotides, which are enriched at genomic regulatory regions, including gene promoters [1,2]. DNA methylation plays a key role in regulating gene expression by recruiting chromatin remodeling complexes or by regulating the binding of transcription factors to the DNA [3].

The methylation status of each CpG site is the result of two counteracting processes.

The gain of DNA methylation is due to the activity of the DNA methyltransferases (DNMTs), which transfer the methyl group from the S-Adenosyl L-Methionine (SAM) to unmethylated cytosines. In particular, DNMT1 is proposed as the maintenance methyltransferase, since it preferentially binds and modifies hemimethylated CpGs originating from DNA replication, thus ensuring the stable transmission of the DNA methylome during cell divisions. The de novo DNA methylation is instead mainly attributed to DNMT3a and DNMT3b, which bind both the hemimethylated and the unmethylated cytosines [2,3].

The methylated cytosines (**5**-methyl-cytosines; 5mC) can be reverted to their unmodified status through passive or active demethylation. The passive process is due to the occasional failure of methylation maintenance during DNA replication. In proliferating cells this can result in a slight, progressive DNA hypomethylation. The active demethylation involves the enzymes from the ten-to-eleven translocation (TET) family. Using Fe (II) and **2**-oxoglutarate (**2**-OG) as cofactors, TETs sequentially oxidize the 5mC to **5**-hydroxy-methyl-cytosine, **5**-formyl-cytosine, and **5**-carboxyl-cytosine. These modified bases are then excised from the DNA by the stepwise action of the thymine–DNA–glycosylase (TDG) and the Base Excision Repair (BER) [4,5,6].

Much attention has been dedicated in understanding how the methylation status of a CpG can be influenced by the status of the neighboring cytosines. Currently, DNA methylation is mainly considered a cooperative process, meaning that the higher the number of 5mCs in a region, the higher the probability is of a cytosine being methylated. In support of this hypothesis, it has been reported that nearby CpGs often share a similar methylation status [7,8,9,10,11]. This pattern of co-methylation has been attributed to the processivity of DNMT3s that, by binding to the DNA as oligomers, simultaneously methylate different nearby cytosines. Moreover, it has been proposed that the hemimethylated cytosines generated by DNMT3s can directly recruit DNMT1 during the de novo methylation, with a consequent spreading of methyl deposition on the surrounding cytosines [12]. Evidence of the cooperativity of DNA methylation can be also derived from mathematical models. Indeed, schemes that do not consider the methylation of a CpG to be dependent on the methylation of the surrounding sites are mostly unable to predict complex patterns of DNA methylation [13,14,15].

Whether DNA demethylation is a cooperative phenomenon is more controversial. It has been shown that TETs mostly oxidize 5mC in a non-processive way, stalling on the DNA after the formation of 5hmC. The resulting accumulation of this base prevents the de-novo DNA methylation and can lead to passive demethylation [4,5,6]. However, on a small fraction of genomic targets, mainly located in regulatory regions, TETs can oxidize 5mCs in a processive way, resulting in an active erasure of DNA methylation [4]. Whether TETs can be preferentially recruited at unmethylated regions is also unclear. Indeed, TETs are mostly indirectly recruited at specific target regions by interacting with other proteins, including transcription factors (TFs) [16]. Nonetheless, TFs can, in turn, bind the DNA in a methylation-sensitive way [17]. Furthermore, it has been proposed that some of the TETs can directly bind stretches of unmethylated cytosines via the CXXC domain. This is thought to be relevant for preventing aberrant DNA methylation at the CpG islands [18].

Recent evidence suggests that methylating and demethylating enzymes act synergistically in shaping the DNA methylation landscape. In particular, Gu et al. demonstrated that DNMTs and TETs contemporarily bind to the DNA, although in a complementary way. The binding of TETs to the gene promoters may avoid the spreading of DNA methylation to the active promoters by confining the DNMT3s outside these regulatory regions [19]. 

Recently, Rulands et al. provided evidence that DNA methylation and demethylation can occur sequentially on the same DNA molecule. By using a modeling approach, they showed that the co-expression of DNMTs and TETs in a cell can result in an oscillatory dynamic, with cytosines being continuously methylated and demethylated. The different kinetics of the methylation and demethylation reactions ultimately produce heterogeneous methylation profiles among cells [20]. Such heterogeneity, with different cells from the same tissues showing different methylation profiles at the same locus, has been extensively described in other studies [21,22,23,24]. 

Despite the presence of this heterogeneity among cells, we and others described that the DNA methylation profiles are mostly conserved when comparing different individuals, suggesting that the cell population as a whole achieves a stable equilibrium in a tissue [25]. 

Mathematical modeling has been extensively applied to DNA methylation and has provided useful insights on the regulatory mechanisms of this process. In a previous study, we used Markov’s hidden chains to model the DNA methylation changes of the D-aspartate oxidase gene promoter during mouse development [26]. The model was, indeed, aimed at capturing the dynamic of DNA methylation over time. In this model, the methylation status of a CpG site was the result of both its intrinsic susceptibility to DNA methylation and of the methylation status of the surrounding cytosines.

In the present study, we developed a new model aimed at analyzing the methylation dynamics, no longer as a function of time, but rather at the steady-state. The model was set up from experimental data obtained by in-depth DNA methylation profiling of 51 samples, using the D-Aspartate Oxidase (DAO) and the D-Serine Oxidase (DDO) as model genes. In this new mathematical model, we took into account both the contemporary effects of methylation and demethylation processes in a region and the influence of neighboring CpGs to these dynamics. 

## 2. Materials and Methods

### 2.1. Ethical Approval

C57BL/6J mice were purchased from the Jackson Laboratory (Bar Harbour, ME). All research involving animals was performed in accordance with the European directive 86/609/EEC governing animal welfare and protection, acknowledged by the Italian Legislative Decree No. 116 (27 January 1992). Animal research protocols were reviewed and consented to by a local animal care committee. 

Human post-mortem brain samples were obtained from The Human Brain and Spinal Fluid Resource Center, Los Angeles Healthcare Center, Los Angeles, CA, USA (cortex and hippocampal samples) and from the MRC London Neurodegenerative Disease Brain Bank of the Institute of Psychiatry, King’s College London, UK (cerebellum samples). All tissues were carried out under the regulations and licenses of the Human Tissue Authority and in accordance with the Human Tissue Act of 2004.

### 2.2. DNA Extraction from Cells and Tissues

The DNA methylation of two genes, the D- Aspartate Oxidase (DDO) and the D- serine Oxidase (DAO), was analyzed, in mouse and human samples respectively. The analysis was performed in different tissues and/or different developmental stages, for a total of 17 different conditions. For each condition, three individuals were analyzed, for a total of 51 analyzed samples. Detailed information about these samples is reported in Table S1a. 

The genomic DNA was extracted from each sample through the Dneasy Blood & Tissue Kit (Qiagen, Hilden, Germany), according to the manufacturer’s instructions. We checked the DNA quality using NanoDrop 2000 (Thermo Scientific, Waltham, MA, USA), and quantified it using Qubit 2.0 Fluorometer (Invitrogen, Carlsbad, CA, USA). 

The sodium bisulfite treatment was performed using the EZ DNA Methylation Kit (Zymo Research, Irvine, CA, USA), following the manufacturer’s instructions. 

A first PCR step was performed using bisulfite-specific primers (Supplementary Table S1b), in the previously described conditions [25]. 

A second PCR step was performed using the Nextera XT primers (Illumina, San Diego, CA, USA), as described in [25]. The amplicons were quantified using Qubit^®^ 2.0 Fluorometer and then paired-end sequenced in 281 cycles per read (281 × 2) using Illumina MiSeq (Illumina, San Diego, CA, USA). The amplicon coordinates and the primers’ sequences are reported in Table S1b.

### 2.3. Sequence Handling

The raw fastq files obtained from the Illumina Miseq platform have been deposited in the European Nucleotide Archive database under the accession numbers PRJEB16320 and PRJEB24382.

Paired-end reads were merged using the PEAR tool [27] with the following parameters: (1) a minimum overlap of 40 bases, (2) minimum read PHRED score of 33. The assembled fastq files were converted into the fasta format through the PRINSEQ tool [28].

For each sample, the DNA methylation profile was analyzed using the AmpliMethProfiler Tool [29]. Reads that were only partially aligned to the amplicon reference, and with more than 98% non-converted cytosines, were discarded by the pipeline. 

The tool returned a CpG methylation profile matrix, where rows represented the retained reads in the fasta file and the columns represented the CpG sites. Each row, indeed, corresponded to the configuration of methylated sites of a single DNA molecule, which will be referred to as epiallele in the following sections.

For each sample, we grouped the epialleles in methylation classes (MCs), based on the number of methylated cytosines they bore. In this way, the epialleles from a region with N CpGs were grouped in N + 1 MCs. The relative frequency of an MC was calculated averaging the number of epialleles in the class for the total number of epialleles obtained for the sample. Finally, considering three different samples for each analyzed condition (gene/tissue/developmental stage), we calculated the average frequency and the standard error of each MC. 

### 2.4. Computations and Statistical Analysis

The rationale and the implementation of the mathematical model will be described in Section 3.2.1 and Section 3.2.2, respectively. All the statistical analyses were performed using R software (version 3.6.0) with an alpha value set for *p* < 0.01.

## 3. Results

### 3.1. In-Depth DNA Methylation Profiling of DAO and DDO Genes

Using high-coverage deep bisulfite amplicon sequencing (HC-ABS) [30,31], we performed an in-depth DNA methylation profiling of two non-housekeeping genes, DDO and DAO, from two different species (mouse and human). For each gene, specific genomic regions near the promoter were chosen, for which we previously had provided evidence of a relationship between the DNA methylation and the gene transcriptional status [25,32]. For the human DAO gene, we analyzed the DNA methylation state at 10 CpG sites (−40:+193) surrounding the transcriptional start site (TSS). For the DDO gene, we analyzed three regions surrounding the TSS: 1) DDO R3 (−903:−531) and DDO R4 (−365:−125), upstream the TSS, bearing 9 and 6 CpGs respectively; 2) DDO R6, downstream the TSS (+295:+540), bearing 6 CpGs. We decided to analyze three sub-regions of the DDO promoter, each one having a limited number of CpGs, to ensure that all the possible epialleles would have been captured during the sequencing procedure. 

For the selected regions, we produced high-coverage amplicon bisulfite sequencing data from several individuals and conditions, obtaining a total of 51 DNA methylation profiles. Detailed information on the analyzed samples is reported in Table S1a. 

We grouped the epialleles from each profile in methylation classes (MCs), based on the number of methylated cytosines they bore. In this way, the epialleles from a region with N CpGs were grouped in N + 1 MCs. We calculated the relative frequencies of each MC by averaging the number of epialleles in the class for the total number of epialleles from the sample. Considering the three different samples for a given condition, we then calculated the average frequency and the standard error of each MC, obtaining the MC distributions (Dn), shown in Figure S1. 

We noted that in all the conditions almost all the possible MCs were represented, although with different frequencies. For example, low methylated classes also had non-zero frequencies in regions with high average methylation and vice versa. 

Figure S1 shows that frequency distributions vary among the analyzed conditions. However, when we compared the MCs distribution of the same condition in different samples, we observed a low interindividual variability, as shown by the error bars in Figure S1. These data suggested that different epigenetic rearrangements take place in each cell but these cell-specific processes seem to achieve a stable and reproducible equilibrium in the cell population as a whole.

**Figure 1:**
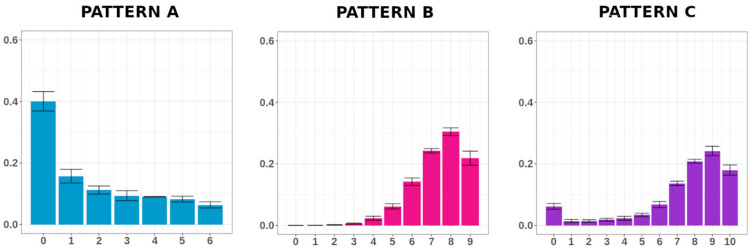
Pattern of methylation class distributions (Dn). Conditions 6, 17 and 1 have been chosen as paradigmatic examples of the Patterns A, B and C, respectively. For each condition, the methylation class (MC) average frequencies and the standard errors are shown.

To simplify the picture and better explore the data, we grouped MC distributions with a similar trend. We individuated three different distribution patterns (Figure 1): (1) Pattern A, shown by regions with a low-MC unimodal trend (e.g., Condition 6), for which the MC distribution (Dn) reached the maximum at a poorly methylated class and gradually dropped; (2) Pattern B, shown by regions with a high-MC unimodal trend (e.g., Condition 17), for which the Dn gradually increased to reach the maximum at high methylated classes; (3) Pattern C, shown by regions with a bimodal trend (e.g., Condition 1), for which the Dn exhibited two peaks at a poorly and a high-methylated class, with one of the two peaks being higher. The groups and the elements that compose them are described in Figure S2. 

Notably, MC distribution was not fully explained by the average methylation of the region, since conditions with similar average methylation exhibited different Dn trends. For example, for Conditions 8 and 11, we calculated an average DNA methylation of 0.25 and 0.29 respectively. However, considering the Dn, Condition 8 exhibited a Pattern A trend whereas Condition 11 exhibited a Pattern B one. 

In summary, the experimental data suggested that: (1) in all the analyzed conditions, a large cell-to-cell heterogeneity of methylation status could be observed; (2) in contrast to this high variability among cells, we observed a low variability between individuals, suggesting the presence of a steady-state equilibrium in the cell population; (3) the different MC distributions could be grouped in recognizable patterns. To explore the dynamics behind these distributions, we developed a mathematical model aimed at reproducing the identified patterns and to get insight into the molecular mechanisms involved. 

### 3.2. Mathematical Modeling of DNA Methylation

#### 3.2.1. Model Assumptions and Rationale

As discussed previously, it seemed to be reasonable to assume that the experimentally observed MC distributions represented the steady-state of inner microscopic dynamics. Assuming the statistical equilibrium means that the timescale for the processes of methylation and demethylation for a single site has to be shorter than the timescales of changes in the external conditions, intended as the concentrations of the methylating and the demethylating enzymes. 

This assumption allowed us to gain information on the inner dynamics by simply comparing its equilibrium distribution with the experimentally observed one. To simplify the dynamics, we adopted a coarse-grained model. This was achieved by using a mean model in which, instead of considering the exact equilibrium distribution for all the possible epialleles, we tried to predict the cumulative distribution of classes of epialleles. 

It was expected that each CpG site had its own methylation or demethylation probability rate, i.e., an intrinsic susceptibility to be methylated or demethylated, independently of the status of the surrounding region. As a consequence, in a detailed model, the DNA methylation and demethylation can occur with site-specific probabilities. We instead adopted a mean model, thus schematizing a mean probability of methylation per unit time P, and a mean probability of demethylation per unit time Q, for each unmethylated and methylated site, respectively. 

A critical aspect that needed to be accounted for was the cooperativity of DNA methylation and demethylation. While the former was expected, as previously described, and had already been analyzed in a microscopic form [33], the latter was less clear. We decided to model both DNA methylation and demethylation as cooperative processes, putting the per-site rate of the methylation and demethylation in linear dependence on the number of methylated and unmethylated cytosines in the region, respectively. This approach allowed us to explore the relative contribution of the fixed and cooperative methylation and demethylation in determining the overall MC distributions.

#### 3.2.2. Model Implementation

To describe the equilibrium conditions dynamically occurring between methylation and demethylation processes, let us consider C different cells, from which the DNA region, containing N CpGs, has been analyzed. The quantity C_n_ denotes the number of such cells having n methylated sites, namely the number of individuals in the n-th class, where n belongs to the interval [0, N]. By definition

(1) $$\overset{N}{\underset{n=0}{\sum }}C_{n}=C$$

Let us assume for simplicity that the transition processes mainly occur between individuals belonging to adjacent classes, namely C_n_ -> C_n+1_ or C_n+1_ -> C_n_. In this case, the equilibrium condition is reached when the law of detailed balance is satisfied, namely

(2) $$C_{n}\left(N-n\right)P_{n}=C_{n+1}\left(n+1\right)Q_{n+1}with\;n\;=\;0,\ldots N-1$$

where P_n_ (and Q_n_) denotes the rate of methylation (and demethylation) per single site, namely the number of transitions for unit of time per single site. To properly describe the quantities P_n_ and Q_n_, a few comments are needed. We modeled the susceptibility to methylation or demethylation in terms of two contributions. An own methylation or demethylation probability rate, independent of the configuration of the rest of the region, and averaged on all CpG sites, here denoted by P and Q correspondingly. In addition to this term, we described the presence of cooperative methylation and cooperative demethylation by introducing a linear dependence of the probability rate per site on the total number of methylated and unmethylated sites in the epiallele, respectively. As a consequence of the previous considerations, we can write

(3) $$P_{n}=P+\alpha n$$

and

(4) $$Q_{n}=Q+\beta \left(N-n\right)$$

where P and Q represent the independent terms whilst α and β stand for the cooperative contributions of the two processes.

**Figure 2:**
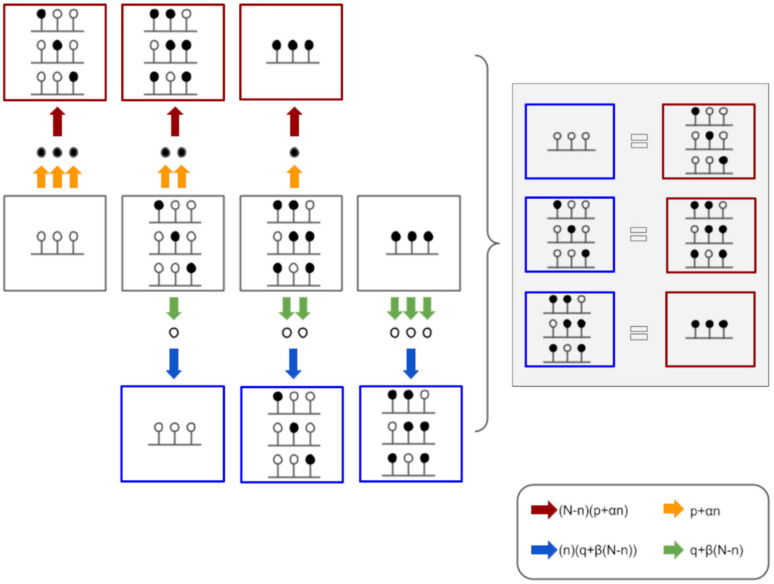
Schematic overview of the model. Left side: for a locus with three CpG sites, the eight different epialleles can be grouped in four MCs, based on the number of methylated cytosines (n). In a dynamic equilibrium, each CpG site can be either methylated (gold arrows) or demethylated (green arrows). The probability of the two processes occurrence is affected by the number of methylated (n) or demethylated (N-n) neighboring cytosines. The probability of the region being methylated (red arrows) or demethylated (blue arrows) depends on the number of cytosines that can be modified. Indeed, the more a region is methylated, the higher is the rate of methylation of a single CpG site, but lower is the number of cytosines that can be methylated. In this scheme, the epialleles produced during the methylation process are circled in red, while those produced during the demethylation process are circled in blue. Right side: Under the steady-state assumption, the number of molecules belonging to the n MC becoming n+1 methylated is equal to the number of molecules belonging to the n+1 MC becoming n methylated.

A schematization of the model just described is offered in Figure 2 for a three-site region.

As stated previously, in our case C constitutes the total number of different cells from which the DNA region has been analyzed. The statistical analysis which we performed can now be specified. For a sample of data coming from the same region of the DNA and from cells from the same tissue, we averaged the experimental distributions of methylated sites for a number of different individuals, depending on the availability of the data, to obtain an average distribution, D_n_. As uncertainties on these values, we adopted the standard errors coming from the fluctuations of the different individuals around the mean value. We then compared the expected distribution, C_n,_ as a function of the parameters P, α, Q, and β via Equation (2), with the experimental distribution through a likelihood:

(5) $$L=\overset{N}{\underset{n=0}{\sum }}\frac{{\left(D_{n}-C_{n}\left(P,\alpha ,Q,\beta \right)\right)}^{2}}{\sigma_{n}^{2}}$$

A point to be noted here is that, we didn’t take into account the intrinsic Poisson fluctuations in determining the uncertainties on the theoretical expectations, since we expected them to be smaller than the statistical uncertainties on the data. Indeed, for each individual, the random fluctuations of the parameters P, α, Q, and β would have induced distributions fluctuating more than the Poisson expectations.

The maximization of the likelihood provided us with the expected values of the parameters. Furthermore, since the likelihood can be well approximated by a Gaussian in the proximity of the maximum, we could produce the 68% confidence levels of the parameters’ central values.

#### 3.2.3. Results Obtained by the Model

**Figure 3:**
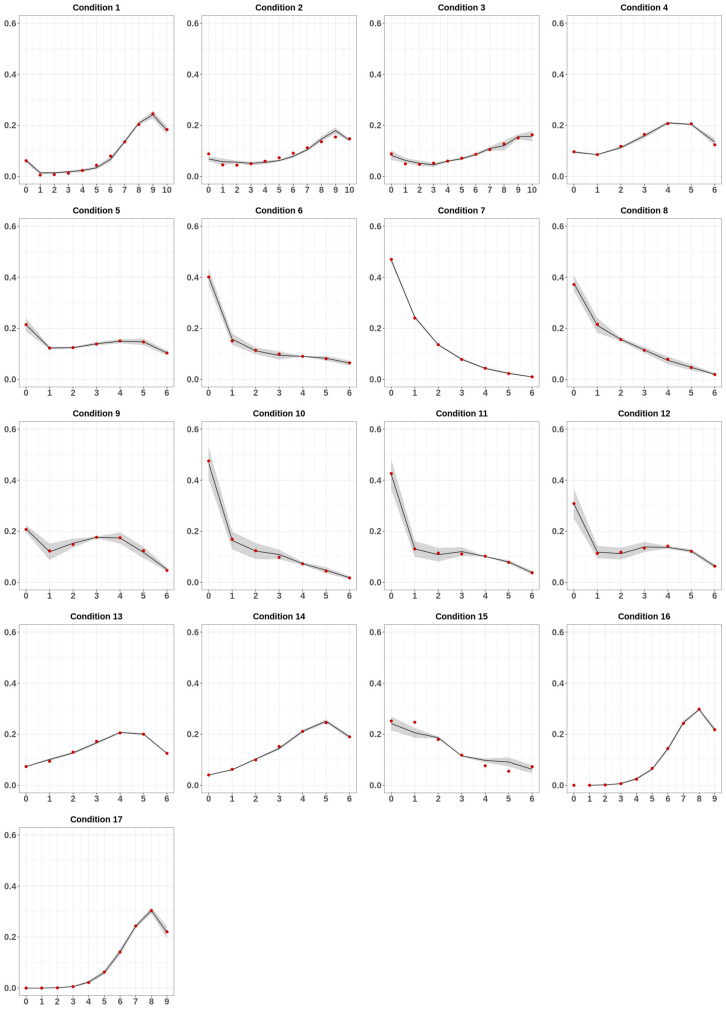
Fit results. For each condition, the experimentally determined average MC frequencies are indicated as red dots. The black lines and the blurred area indicate the average MC frequencies and its respective error predicted by the model.

In order to measure the prediction capabilities of our model, we compared the experimental data with their theoretical predictions (Figure 3). In Figure 3, the red dots represent the observed average MC frequencies, whereas the black lines and the blurred areas indicate the theoretical predictions of the model and their error. Interestingly, at a glance, the predictions generally fitted extremely well the data. In support of this observation, the reduced-χ^2^ values for the analyzed conditions showed an agreement of the fit with the experimental data well inside of 1-σ statistical uncertainty (Table S2). 

For the few cases in which this did not occur, a check with the corresponding panel of Figure 3 showed that there were very few points not matching the data, with an overall behavior well captured by the model. In Table S3 we reported the best fit values for the free parameters P, α, Q, and β for all the analyzed datasets. 

Comparing the central determination for P, α, Q, and β with their variance (Table S3), it could be observed that in all the cases the parameters’ central values were not compatible with a zero value at 2-σ level, meaning that all of the parameters were statistically necessary in the description. This suggested that the model had the statistical power to determine its parameters, and that it was not overfitting the data.

Some of the conditions we studied were made up of the same genomic region and the same tissue, but at different stages of development. It is worth observing that, in these cases, the values of P, α, Q, and β of the different developmental stages were very similar among each other. This seemed to suggest that such values were mainly determined by tissue and gene-specific characteristics, and were less dependent on the developmental stage. 

**Figure 4:**
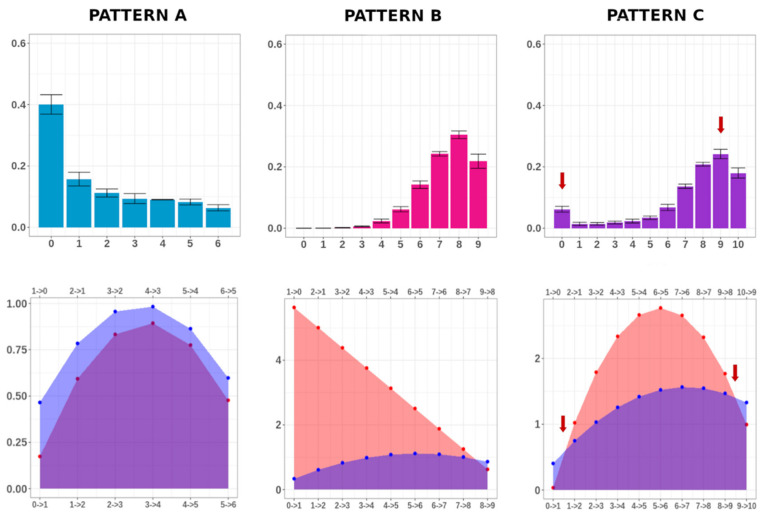
MC frequency distribution and methylation/demethylation rate comparison. For Conditions 6, 17, and 1, the MC average frequencies and the respective standard error (calculated on three individuals) are indicated in the upper panels. For the same conditions, the methylation (red) and demethylation (purple) rates are shown in the lower panels. For Condition 1, DNA methylation and demethylation occur at quite the same rate for molecules with 0 and 9 methylated cytosines (red arrows, lower panel). Notably, these two MCs exhibited the highest average frequency (red arrows, upper panel).

In Figure 4, using the three examples reported in Figure 1, we compared the pattern of MC distribution (upper panels) with the rate of methylation and demethylation per MC (lower panels), calculated from Equation (2), which regulates the transition of the epialleles between adjacent MCs. For Patterns A and B, the probability rate of one of the processes (DNA demethylation for Pattern A and DNA methylation for Pattern B) dominated the other one. The MC distributions for the corresponding conditions were indeed unimodal, and peaked at the fully-unmethylated class (e.g., Condition 6) or at the maximally-methylated class (e.g., Condition 17). Differently, for Pattern C, the quantities (N − n) P_n_ and (n + 1) Q_n+1_ equilibrated at two particular MCs, indicated by the red arrows (lower panel). In this case, as it can be easily seen from the characteristics of Equation (2), the MC distribution showed a bimodal behavior, with two peaks (indicated by the red arrows in the corresponding upper panel). 

To estimate the contribution of the fixed and cooperative methylation and demethylation in determining the overall MC distributions, we calculated the ratio between the cooperative and the independent parameters’ values for DNA methylation (α/P) and DNA demethylation (β/Q). We found that the cooperative term overcame the independent counterpart in almost all the analyzed regions, for both DNA methylation and demethylation (mean α/p = 4, standard deviation = 7.24; mean β/q = 0.52, sd = 0.63). We only observed three conditions deviating from this rule. Indeed, for the Conditions 16 and 17, the independent methylation parameter, P, resulted in the major contributor to the observed MC distributions. It is worth noting that, in these two cases the analyzed region was placed at a distance from the gene TSS compatible with that described by GU et al. [19] as the one with the highest bond with DNMTs. We also observed a prevalence of independent DNA demethylation in Condition 15.

**Figure 5:**
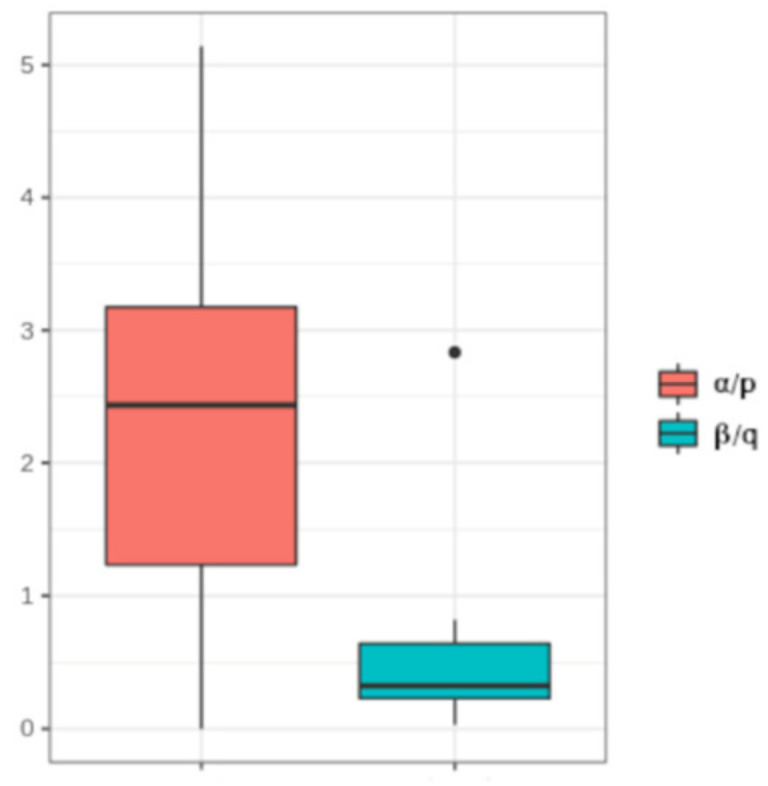
Distribution of the methylation and demethylation ratios. Boxplot of the DNA methylation ratio (α/p) and of the DNA demethylation ratio (β/q) in the analyzed samples.

Finally, we estimated whether the cooperative effect was more important in methylation or demethylation. As shown in Figure 5, the methylation and demethylation ratios exhibited different inter-sample distributions (W = 243, *p*-value = 0.00041). In particular, we observed higher ratios for DNA methylation. This suggested a higher dependence of the methylation probability rate on the overall methylation of a molecule than for DNA demethylation.

## 4. Discussion

In this study, we analyzed DNA methylation at a single molecule level, by performing deep amplicon bisulfite sequencing. Using a modeling approach, we explored the DNA methylation and demethylation dynamics in these genomic regions. We also investigated how the methylation status of neighboring CpGs can affect the probability of a cytosine to be methylated or demethylated. 

When analyzing the experimentally-determined DNA methylation profiles, we found that almost all the possible methylation classes were represented in all the tested conditions, thus suggesting that the methylation status of a certain locus differed from cell to cell. We had already observed a similar cell-to-cell heterogeneity in a previous study [25], and excluded that this heterogeneity derived from the cell-type composition of a tissue. Furthermore, stochastic variations in epiallelic profiles were also described by other authors using deep bisulfite sequencing [24], long reads sequencing [23], and single-cell bisulfite sequencing [21,22]. 

In contrast to this heterogeneity among cells, we observed a low variability in the MC profiles of the same region and the same tissue, from different individuals. Also, this finding was consistent with the results from our previous papers, obtained from fewer samples [26,33].

Taken together, the experimental data suggested that the observed MC distributions could reflect a statistical equilibrium between DNA methylation and DNA demethylation. The co-occurrence of these two processes in a DNA region was also explored by Rulands et al. [20] and it was, partially, supported by evidence of the contemporary binding of methylating and demethylating enzymes to the DNA, provided by Gu et al. [19].

To explore the possible variables involved in this balance, we developed a mathematical model by assuming that the methylation status observed at each CpG on a generic DNA region reflected the steady-state of inner microscopic dynamics involving DNA methylation and demethylation. The main idea behind such an approach was that the timescale for the two processes was shorter than the timescale for changes in the external conditions (here intended as the change in the concentrations of the enzymatic machineries). This ensures that the kinetics of DNA methylation, and demethylation of individual sites, can reach the statistical equilibrium before external conditions relevantly change.

In order to reduce the statistical noise of the samples, we adopted a coarse-grained description of the DNA methylation profiles, that is, instead of considering the exact equilibrium distribution for all epialleles, we considered the cumulative distribution of classes of epialleles (MCs), defined by the number of methylated CpGs they harbored, as introduced in [26]. In this framework, analogously to Affinito et al. [26], we adopted the simplicity ansatz that the epiallele transitions involve only adjacent MCs, which means that in the unit time only one of the cytosines of an epiallele can change its status and become methylated or demethylated. According to our model, the equilibrium condition is reached when the methylation rate of a generic MC n is equal to the demethylation rate of the corresponding n + 1 MC. This is a key difference in respect of the approach we previously adopted [26], where the epialleles’ transitions were modeled as out-of-equilibrium dynamics.

In our previous paper [33], modeling DNA methylation as a cooperative process proved to be crucial to fit the experimental data. In the present analysis, we hence took profit from this observation and modeled the methylation probability rate of a CpG as linearly dependent on the number of methylated cytosines in the epiallele. In this new model, we have also added the possibility of cooperative effects in the demethylation processes.

When comparing the predictions from this very simple model with the experimentally-observed MC distributions, we observed a very satisfactory agreement, as proved by the reduced-χ2, which furthermore guaranteed that the model was not overfitting the data. 

The first conclusion that we can draw from these results is that the experimental data were compatible with the co-occurrence of DNA methylation and demethylation at the same genomic locus. Of note, a similar equilibrium between gain and loss of DNA methylation was proposed as a mechanism of epigenetic memory during the exit from pluripotency [34]. We demonstrated that these dynamics can be observed also in adult, non-proliferating, cells. 

Moreover, the values found for the parameters of the model were quite homogeneous for conditions sharing the same gene and tissue, thus supporting the idea of a good level of generality of the description. Finally, the confidence level of the parameters describing the methylation/demethylation probability rates were all statistically relevant (not compatible with zero at 2σ level). These results suggest that the cooperative effects cannot be neglected, both for DNA methylation and demethylation. The latter can both reflect the refractoriness of low methylated regions to be methylated, or be the result of the preferential binding of TETs to unmethylated CpGs, and of their processivity [4,18]. Furthermore, the model parameters suggested that the cooperative effect of DNA methylation was in almost all the cases the main contributor to the methylation probability rate of cytosines. On the contrary, the cooperative term of DNA demethylation, even though relevant for the description, was never dominant.

We have to point out now some limitations of the present study. First, we analyzed a limited number of samples (n = 3) for each condition. However, a more robust estimation of the average MC distributions and their related uncertainties would require a larger sample cohort. Second, we analyzed a limited number of target regions. Indeed, we cannot generalize the equilibrium dynamics here described to all the genome. Further studies are needed to understand if these dynamics also apply to other genomic regions. However, this loss of generalization is balanced, in our opinion, by a gain in statistical power. Indeed, the high sequencing depth can more efficiently capture the cell-to-cell heterogeneity in DNA methylation profiles. Although we tested various tissues and organisms in this study, it will be necessary to expand these observations to further organisms and tissues to achieve an even higher generalization. Finally, in the present study, we also analyzed conditions sharing the same gene and tissue at different developmental stages. Since each stage was considered as an independent condition, we could conclude that our model could be generalized to different developmental stages. However, due to the limited number of time-points, we are unable to state firm conclusions on this aspect.

Concerning the mathematical description, a limitation is certainly represented by the coarse-grained description, in terms of methylation classes. In fact, if it represents a good trade-off between the dimension of the data sets and complexity of the model, it does not allow scrutinization of the peculiar characteristics of each CpG, in terms of peculiar probability of methylation or demethylation of that site.

## 5. Conclusions

In the present study, we analyzed the DNA methylation of two loci at the single-molecule level in several individuals and conditions. We then developed a mathematical model taking into account cell-to-cell heterogeneity in the DNA methylation status, and the inter-individual stability of DNA methylation profiles, observed in the experimental data. The good agreement between the experimental observations and the model theoretical predictions suggested a potential co-occurrence of DNA methylation and demethylation in the same genomic region. Furthermore, the model parameters suggested that the rate of both processes is relevantly influenced by the methylation status of the surrounding region. Further experiments will be required to validate our model, as well as to investigate the molecular mechanisms that underlie these phenomena.

## Supplementary Materials

The following are available online at https://www.mdpi.com/2218-273X/10/9/1271/s1, Figure S1: Per-sample MC distributions, Figure S2: Average MC distributions of the analyzed conditions grouped by the pattern, Table S1a: Detailed information about the analyzed conditions, Table S1b: Amplicon coordinates and primers, Table S2: Reduced χ2 values, Table S3: Model free parameters’ values and variance.
