# Supplementary material for: Modeling DNA Methylation Profiles through a Dynamic Equilibrium between Methylation and Demethylation
Source: Biomolecules. 2020 Sep 3;10(9):1271. doi: 10.3390/biom10091271 (PMC7564540; doi:10.3390/biom10091271)
Supplement: Supplementary file 1 [file biomolecules-10-01271-s001.pdf]

biomolecules

Article

Supplementary

Modeling DNA Methylation Profiles through a Dynamic Equilibrium between Methylation and Demethylation

Giulia De Riso [1,*,†], Damiano Francesco Giuseppe Fiorillo [2,3,†], Annalisa Fierro [4], Mariella Cuomo [1,5], Lorenzo Chiariotti [1,5], Gennaro Miele [2,3] and Sergio Cocozza [1]

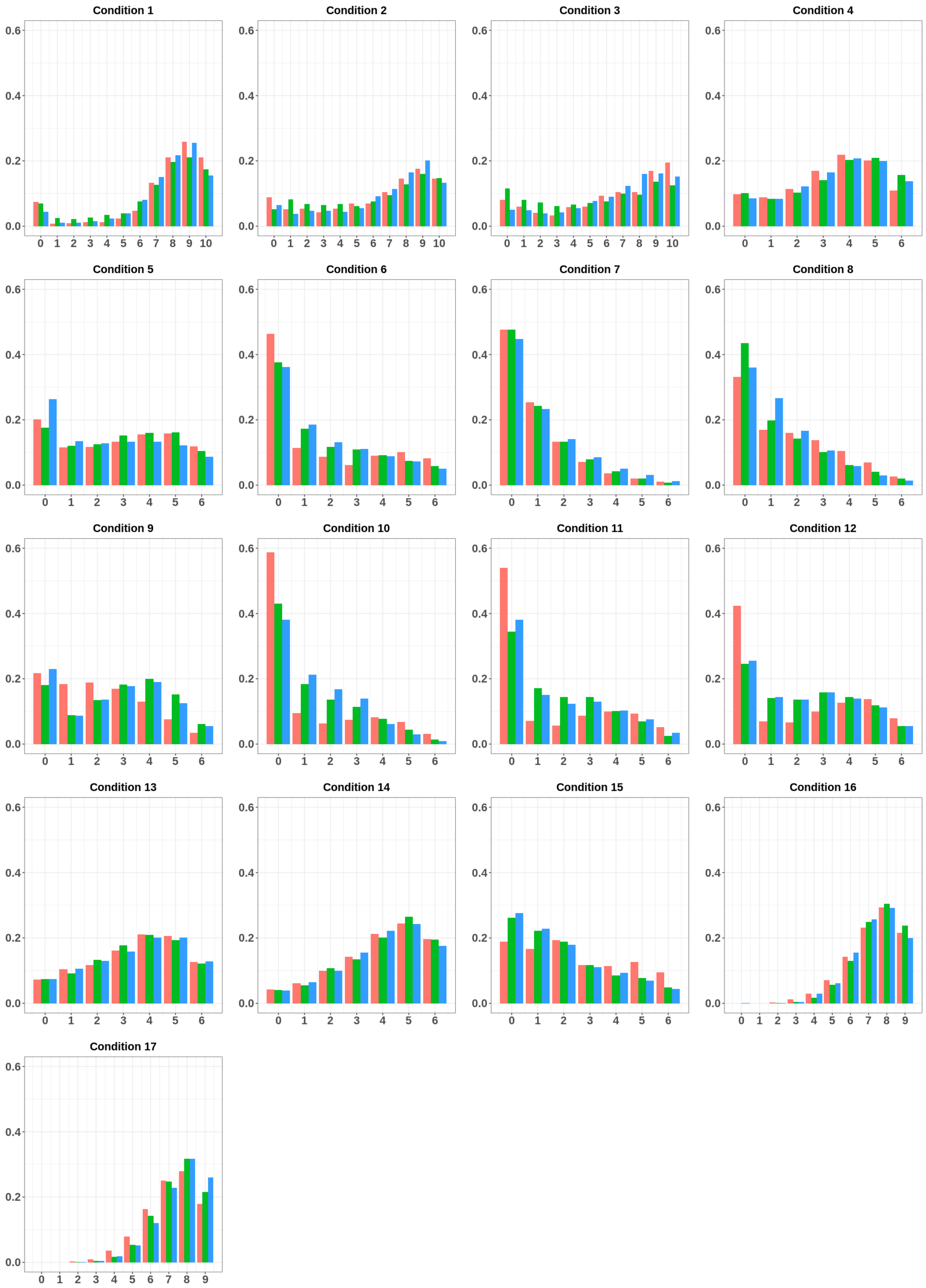

Figure S1. Per-sample MC distributions.The number of epialleles for the different Methylation Classes is shown for three samples (sample 1 in pink, sample 2 in green, and sample 3 in blue).

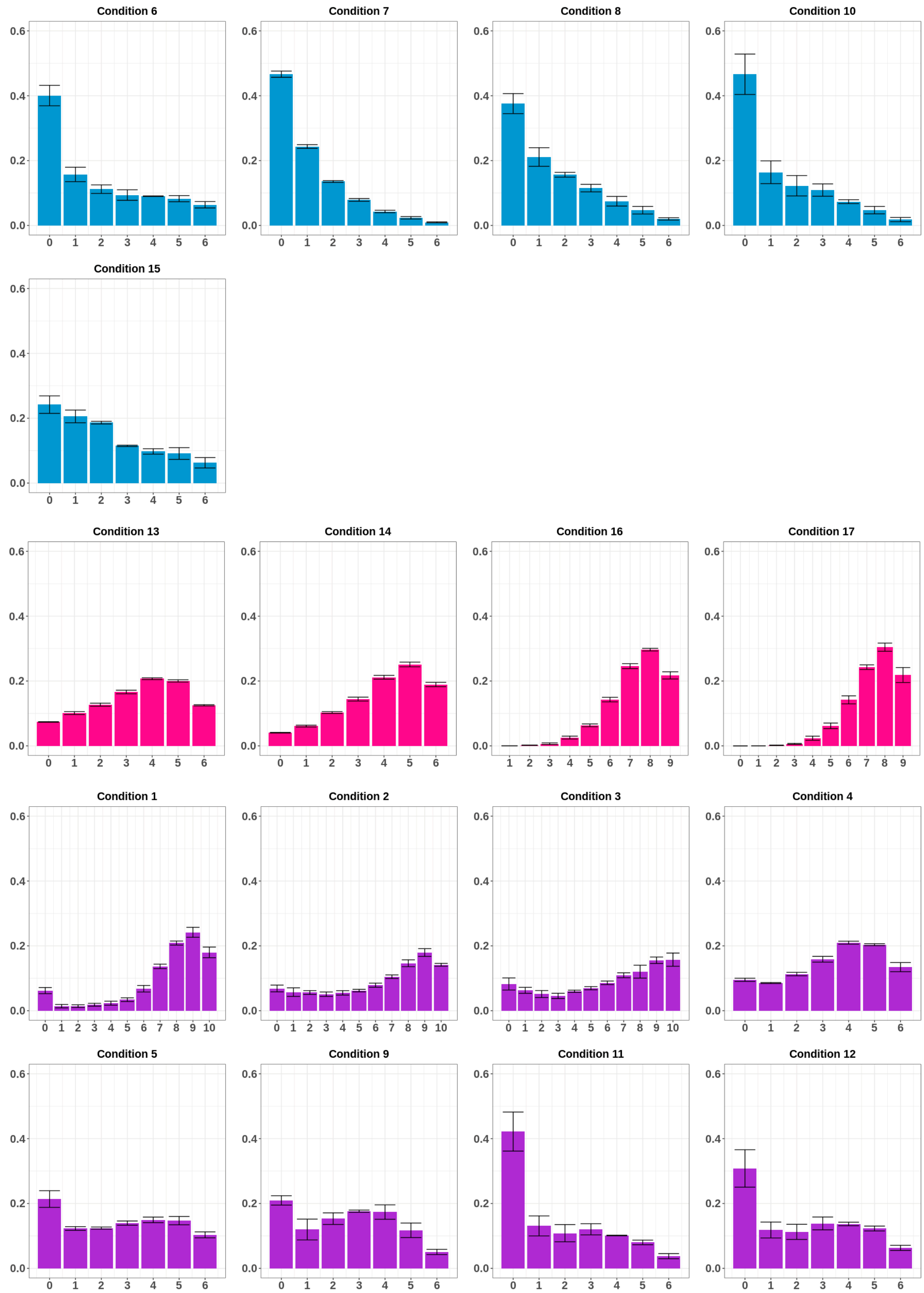

Figure S2. Average MC distributions of the analyzed conditions grouped by the pattern.

Table S1a.: Detailed information about the analyzed conditions.

Sample	Organism	Gene	Tissue	Developmental Stage	CpGs	Average Methylation	AN
Condition1	human	DAO	Cerebellum	Post-mortem	10	0.74	PRJEB24382
Condition2	human	DAO	Cortex	Post-mortem	10	0.63	PRJEB24383
Condition3	human	DAO	Hippocampus	Post-mortem	10	0.61	PRJEB24384
Condition4	mouse	DDO R4	Whole brain	E15	6	0.58	PRJEB16320
Condition5	mouse	DDO R4	Whole brain	P0	6	0.46	PRJEB16321
Condition6	mouse	DDO R4	Whole brain	P30	6	0.3	PRJEB16322
Condition7	mouse	DDO R4	Gut	P0	6	0.18	PRJEB16323
Condition8	mouse	DDO R4	Gut	P15	6	0.25	PRJEB16324
Condition9	mouse	DDO R4	Gut	P90	6	0.42	PRJEB16325
Condition10	mouse	DDO R4	Lung	P0	6	0.23	PRJEB16326
Condition11	mouse	DDO R4	Lung	P15	6	0.29	PRJEB16327
Condition12	mouse	DDO R4	Lung	P60	6	0.38	PRJEB16328
Condition13	mouse	DDO R6	Brain	E15	6	0.57	PRJEB16329
Condition14	mouse	DDO R6	Brain	P0	6	0.66	PRJEB16330
Condition15	mouse	DDO R6	Brain	P30	6	0.36	PRJEB16331
Condition16	mouse	DDO R3	Brain	P0	9	0.82	PRJEB16332
Condition17	mouse	DDO R3	Brain	P30	9	0.82	PRJEB16333

*DAO: D-serine Oxidase; DDO R3: D-Aspartate Oxidase Region 3; DDO R4: D-Aspartate Oxidase Region 4; DDO R6: D-Aspartate Oxidase Region 6; AN: Accession Number of the condition's raw data in the European Nucleotide Archive database.

Table S1b. Amplicon coordinates and primers,.

Gene	Amplicon Coordinates	Genome Assembly	Primer FW	Primer RV	Size Amplicon (bp)
*DDO R3	CHR10:40629084-40629511	GRCm38/mm10	GttttTTaTatgtTttggagTTt	acctccctAaaaAtcatttAattcta	427
*DDO R4	CHR10:40629543-40629947	GRCm38/mm10	gtgtgtttTtgaggaggtgaTaTtTa	aActtaccctccattAAtccatAcc	405
*DDO R6	CHR10:40630278-40630681	GRCm38/mm10	TttagtgttaaTttattagagTtgtgg	AatacaatcccttcttAcaacaAAca	403
*DAO	CHR12:108879923-108880252	GRCh38/hg38	aaggTTtgtTTaTaggggTttgaga	ccaActcaaaAAtAcatctAccactc	329

*DAO: D-serine Oxidase; DDO R3: D-Aspartate Oxidase Region 3; DDO R4: D-Aspartate Oxidase Region 4; DDO R6: D-Aspartate Oxidase Region 6.

Table S2. Reduced $\chi 2$ values.

Sample	red.-$\chi 2$
Condition1	2.4
Condition2	6.08
Condition3	0.78
Condition4	2.98
Condition5	0.07
Condition6	0.27
Condition7	0.13
Condition8	0.16
Condition9	0.4
Condition10	0.28
Condition11	0.38
Condition12	0.19
Condition13	4.5
Condition14	3.92
Condition15	20.5
Condition16	0.9
Condition17	0.35

Table 3. Model free parameters' values and variance.

Sample	P	σP	α	σα	Q	σQ	β	σβ
Condition1	0.0035	0.0003	0.11	0.0012	0.133	0.002	0.0302	0.0007
Condition2	0.0338	0.0013	0.1	0.0005	0.0941	0.0009	0.063	0.0005
Condition3	0.038	0.002	0.09	0.0009	0.082	0.002	0.0672	0.0008
Condition4	0.0437	0.0009	0.1	0.0006	0.1571	0.001	0.0279	0.0004
Condition5	0.0368	0.0014	0.1	0.002	0.126	0.003	0.0521	0.0016
Condition6	0.029	0.0003	0.09	0.0004	0.0996	0.0011	0.0731	0.0003
Condition7	0.0484	0.0006	0.06	0.0009	0.135	0.003	0.0866	0.001
Condition8	0.049	0.002	0.08	0.002	0.179	0.006	0.065	0.002
Condition9	0.0285	0.001	0.11	0.002	0.249	0.004	0.0078	0.001
Condition10	0.0274	0.0014	0.09	0.002	0.212	0.006	0.05	0.0019
Condition11	0.0199	0.0004	0.1	0.0008	0.1826	0.0019	0.0413	0.0006
Condition12	0.0205	0.001	0.11	0.0015	0.175	0.003	0.0321	0.0011
Condition13	0.0747	0.0002	0.1	0.0002	0.1491	0.0003	0.04034	0.00013
Condition14	0.08267	0.00008	0.1	0.00006	0.12281	0.00011	0.03981	0.00004
Condition15	0.0918	0.0012	0.04	0.0013	0.037	0.003	0.1048	0.0011
Condition16	0.6155	0.0019	0	0.0005	0.0969	0.0006	0.03202	0.00018
Condition17	0.626	0.009	0	0.0016	0.096	0.002	0.0301	0.0011
